# Outcomes and Prognostic Factors of Metastatic Gastric Cancer: A Single-Center Experience

**DOI:** 10.7759/cureus.28426

**Published:** 2022-08-26

**Authors:** Mohamed Aseafan, Ahmed Mostafa Gad, Bader Alshamsan, Naela Agha, Ali Alhanash, Ali H Aljubran, Ahmed Alzahrani, Shouki Bazarbashi

**Affiliations:** 1 Oncology Centre, King Faisal Specialist Hospital and Research Centre, Riyadh, SAU; 2 Section of Medical Oncology, Department of Internal Medicine, Security Forces Hospital Program, Riyadh, SAU; 3 Clinical Oncology and Nuclear Medicine Department, Faculty of Medicine, Ain Shams University, Cairo, EGY; 4 Department of Medicine, College of Medicine, Qassim University, Qassim, SAU; 5 Oncology Centre, King Faisal Specialist Hospital and Research Centre, Ryiadh, SAU; 6 Oncology Department, King Khalid University Medical City, Abha, SAU

**Keywords:** gastroesophageal cancer, progression-free survival, cancer survival, metastatic gastric cancer, gastric cancer

## Abstract

Background

Gastric cancer (GC) carries a poor survival outcome despite the availability of many therapeutic agents active in treatment. In this study, we aimed to evaluate the survival outcomes of metastatic GC treatment from a single center in Saudi Arabia and identify possible prognostic factors.

Methodology

Data on patients diagnosed with metastatic GC between December 2009 and November 2013 were collected and analyzed.

Results

During this period, 41 patients were diagnosed with a median age at diagnosis of 52 years, and 56.1% of patients were males. Only four (9.2%) patients had human epidermal growth factor receptor 2 overexpression. Overall, 83% were treated with oxaliplatin-based chemotherapy. The median progression-free survival (PFS) and overall survival (OS) were 4.1 and 15.4 months, respectively. Female sex was an independent prognostic factor for better PFS and OS. Normal lymphocyte count was associated with improved PFS.

Conclusions

Our study highlights poor outcomes in patients with metastatic GC and the need for further research in this field.

## Introduction

Gastric cancer (GC) is the fifth most common cancer and the fourth most common cause of cancer-related death worldwide [1]. Due to a lack of early symptoms, GC often presents in an advanced stage, characterized by poor survival [2]. The survival rates for advanced GC are among the worst of most solid tumors, with a median survival time of four months without systemic chemotherapy [3].

Systemic therapy has been shown to extend survival compared to the best supportive care in the advanced stage [4-7]. Despite the availability of many new chemotherapeutic, biological, and immune targeting agents, the outcome continues to be poor for patients with metastatic disease, with median overall survival (OS) of 8-16 months.

The management of patients with metastatic GC continues to be a challenging process. There is no standard first-line chemotherapy regimen, and several options are available and considered acceptable. First-line therapy usually consists of fluoropyrimidine and a platinum compound. However, some regimens incorporate irinotecan and taxanes in the first-line treatment. Around 20% of patients with GC have human epidermal growth factor receptor 2 (HER-2) overexpression. Adding trastuzumab to first-line, platinum-based chemotherapy in this group of patients has improved survival [4,5]. Recently, checkpoint inhibitors have been incorporated in the first-line setting with improvement in survival [5,7].

Few real-world data address the treatment pattern and outcomes of patients with metastatic GC from Saudi Arabia. In this report, we evaluate the treatment outcomes of patients with metastatic GC treated with systemic therapy at a tertiary care institution in Saudi Arabia. We also discuss the relevant prognostic factors in this group of patients.

## Materials and methods

The medical records of patients diagnosed with metastatic GC between December 2009 and November 2013 were reviewed. Patients were eligible for inclusion if they had (1) histologically confirmed gastric or gastroesophageal junction (GEJ) adenocarcinoma; (2) the presence of distant metastases; (3) age over 14 years (according to the local ministry of health law for adult patients); and (4) received systemic treatment for metastatic disease.

Collected data included age at diagnosis, sex, Eastern Cooperative Oncology Group (ECOG) performance status (PS), histological type, the site and number of metastases, prior surgery and adjuvant chemotherapy, and baseline laboratory findings, including complete blood count and chemistry profile. Data on treatment regimens, including the number of cycles and duration of treatment, and best response, were also collected. All radiological studies were reviewed for assessment of response. Response Evaluation Criteria in Solid Tumors (RECIST v. 1.1) were used to evaluate the response to treatment. OS was calculated from the initiation of systemic treatment to death. Progression-free survival (PFS) was calculated from starting systemic therapy to the date of disease progression or death.

Univariate and multivariate analyses were performed using the Cox regression hazard model. Survival curves were plotted using the Kaplan-Meier method and compared by the log-rank test. A P-value of <0.05 was considered statistically significant.

The study was approved by the hospital Research Ethics Committee (approval number: RAC 2161-128). Given the study’s retrospective nature, the hospital Research Ethics Committee approved a waiver of consent.

## Results

A total of 41 patients with metastatic gastric/GEJ adenocarcinoma who received palliative chemotherapy were eligible for analysis. The median age at diagnosis was 52 years (range = 15-75 years). In total, 23 (56.1%) patients were males, with a male-to-female ratio of 1.27:1. Moreover, 19 (46%) patients had ECOG PS of 0-1. More than half of our patients (53.6%) were adenocarcinoma Not Otherwise Specified (NOS), followed by signet ring differentiation (29.3%) and intestinal subtype in (12.2%). In total, 24 (58.5%) patients had poorly differentiated histological grades, followed by 36.6% with moderately differentiated grades. The most common primary site at diagnosis was distal GC, accounting for 41.5%, followed by GEJ (19.5%). Of note, 58.5% of patients had synchronous metastasis in two or more organs. The most common site of metastasis were lymph nodes (48.8%), followed by the peritoneum (43.9%) and liver (39%). Out of 28 patients tested for HER-2 expression by immunohistochemistry (IHC), four (9.8%) patients had HER-2 overexpression. Patient characteristics are illustrated in Table 1.

**Table 1:** Patient, disease, and tumor characteristics. ECOG: Eastern Cooperative Oncology Group; NOS: not otherwise specified; GEJ: gastroesophageal junction; HER-2: human epidermal growth factor receptor 2 IHC: immunohistochemistry

Characteristics	n (%)
Age (years)	
Median	52
Range	15–75
Sex	
Male	23 (56.1%)
Female	18 (43.9%)
Performance status (ECOG)	
0	2 (4.9%)
1	17 (41.5%)
2	10 (24.4%)
3	5 (12.2%)
4	1 (2.4%)
Unknown	6 (14.6%)
Histological grade	
Moderately differentiated	15 (36.6%)
Poorly Differentiated	24 (58.5%)
NOS	2 (4.9%)
Histological subtype	
Diffuse	2(4.9%)
Intestinal	5(12.2%)
Signet ring	12(29.3%)
Not specified	22(53.6%)
Primary site at diagnosis	
GEJ	8 (19.5%)
Proximal	6 (14.6%)
Distal	17 (41.5%)
Linitis plastica	4 (9.8%)
Not specified	6 (14.6%)
Number of organ involvement	
1	8 (19.5%)
≥2	24 (58.5%)
Site of metastasis	
Liver	16 (39%)
Lung	4 (9.8%)
Bones	3 (7.3%)
Peritoneum	18 (43.9%)
Lymph nodes	20 (48.8%)
Others	6 (14.6%)
HER-2 expression (IHC)	
1+	19 (46.3%)
2+	5 (12.2%)
3+	4 (9.8%)
Not done	13 (31.7%)

Most patients (83%) received oxaliplatin-based, first-line chemotherapy (epirubicin, oxaliplatin, and capecitabine (EOX) 65%, folinic acid, fluorouracil, and oxaliplatin (FOLFOX)/oxaliplatin and capecitabine (XELOX) 17%). Seven (17%) patients received cisplatin-based, first-line chemotherapy (cisplatin-5-fluorouracil (5FU)/capecitabine). Four (9.8%) patients received concurrent trastuzumab. The median number of chemotherapy cycles received was five (range = 1-25 cycles). Overall, 15 (36%) patients received second-line chemotherapy upon disease progression. Monotherapy with docetaxel (14.6%) or irinotecan (17.1%) was the treatment of choice. Doublet oxaliplatin-based chemotherapy was administered to 5% of those patients if not given in the first line. Noteworthy, six (14.6%) patients of the entire cohort had prior surgery, two (4.9%) received prior adjuvant chemotherapy, and one received prior adjuvant chemoradiotherapy.

Our study’s objective response rate (ORR) to first-line therapy was 14.6%, with one patient achieving a complete response (2.4%). Four (9.8%) patients had stable disease, while 26 (63.4%) developed disease progression. The clinical benefit rate (CBR) was 24.4%.

**Figure 1:**
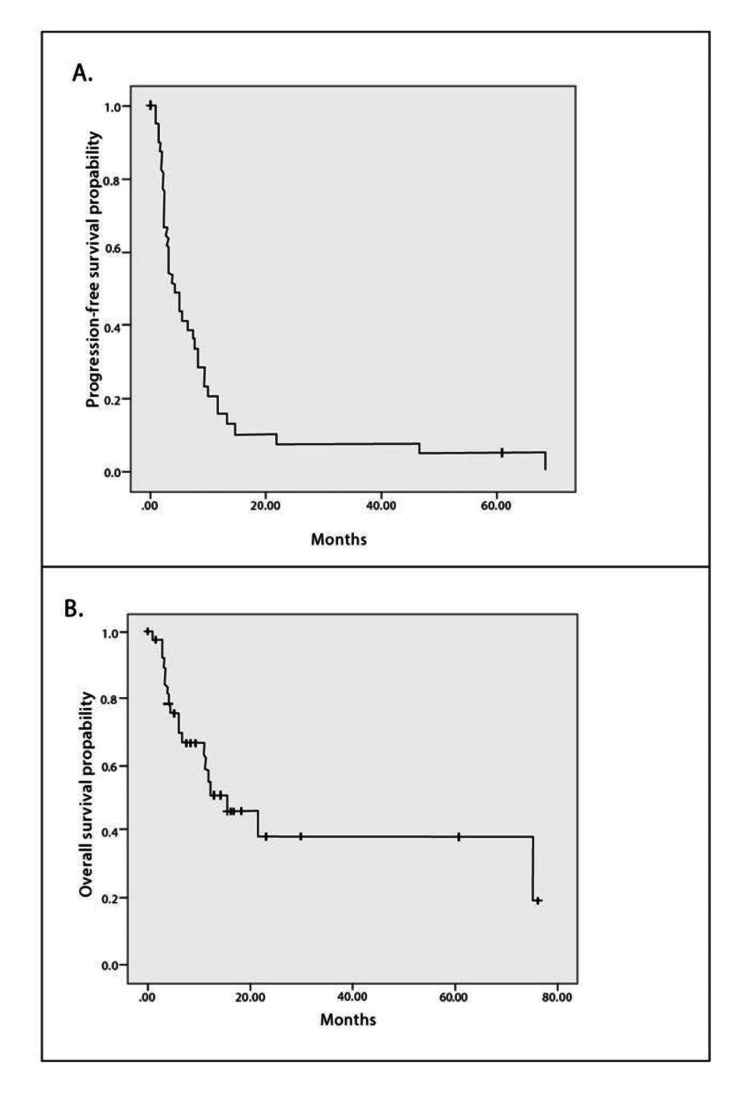
Kaplan–Meier plots of (A) progression-free survival and (B) overall survival (OS) in patients treated for metastatic gastric cancer.

At a median follow-up duration of 15.5 (range = 11.2-19.7) months, the median OS was 15.4 months (95% confidence interval (CI) = 5.9-24.9 months), and the five-year survival was 39%. The median PFS was 4.1 months (95% CI = 1.8-6.3 months) (Figure 1).

Table 2 summarizes the univariate analysis of PFS and OS by different potential prognostic factors. Female sex, having no lymph node metastases, and normal lymphocyte count at baseline were the only factors associated with a statistically significant longer PFS (p = 0.002, 0.014, and 0.005, respectively). Female sex, normal hemoglobin level, and albumin levels at baseline were the only factors associated with improved OS (p = 0.002, 0.046, and 0.003, respectively).

**Table 2:** Univariate analysis of progression-free and overall survival by prognostic subgroup. CI: confidence interval; ECOG PS: Eastern Cooperative Oncology Group performance status; GEJ: gastroesophageal junction; diff: differentiated; Mod: moderately

Variable	Comparator	Progression-free survival (months)			Overall survival (months)		
		Median	95% CI	P-value	Median	95% CI	P-value
Age	<40	2.13	(1.28, 9.7)	0.965	15.40	(2.69, )	0.833
	≥40	4.83	(12.7, 7.56)		11.82	(6.08, )	
Sex	Female	9.2	(4.9, 13.08)	0.002	75.5	(11.2, )	0.002
	Male	2.2	(1.91, 3.19)		06.1	(3.38, 15.4)	
ECOG PS	0–1	4.1	(2.1, 9.7)	0.40	15.40	(4.21, )	0.656
	≥2	4.8	(2.14, 8.18)		21.55	(6.64, )	
Primary site	Distal	6.4	(2.27, 11.5)	0.325	75.5	(3.19, )	0.428
	Proximal/GEJ	4.1	(1.28, 5.6)		8.9	(3.9, 21.60)	
Liver metastasis	Yes	3.18	(2, 4.96)	0.533	11.2	(3.38, )	0.953
	No	5.61	(2.1, 9.23)		15.4	(6.18, )	
Peritoneal metastasis	Yes	6.4	(1.5, 11.53)	0.588	15.40	(9.12, 21.69)	0.931
	No	3.8	(2.73, 5.62)		12.15	(0.00, 25.13)	
Lymph nodes metastasis	Yes	2.9	(1.91, 5.62)	0.014	11.17	(3.9, )	0.327
	No	8.1	(2.14, 11.53)		21.55	(6.08, )	
Numbers of organs involved	1	1.7	(0, 11.53)	0.456	21.55	(0.78, )	0.623
	≥2	4.8	(2.26, 7.56)		15.40	(6.63, )	
Histological grade	Poorly diff.	3.1	(1.84, 6.44)	0.490	15.40	(4.21, )	0.633
	Mod. diff.	6.19	(2.14, 11.53)		21.55	(3.19, )	
Signet ring	Yes	7.3	(0.00, 16.00)	0.289	75.56	(6.08, )	0.581
	No	3.8	(1.47, 6.15)		15.40	(7.69, 23.12)	
Lymphocyte count	Decreased	2.2	(1.6, 2.99)	0.005	NE	(NE, NE)	0.385
	Normal	7.46	(3.8, 9.76)		15.40	(10.8, )	
Hemoglobin level	Decreased	3.1	(2.1, 5.62)	0.188	11.17	(4.01, 21.6)	0.046
	Normal	7.79	(2, 14.72)		75.56	(6.08, )	
Albumin level	Decreased	3.1	(2.14, )	0.302	3.18	(2.96, )	0.003
	Normal	4.1	(2.27, 8.18)		21.55	(10.84, )	
Treatment regimen	Oxaliplatin-based	3.98	(2.26, 7.56)	0.728	12.15	(6.17, )	0.510
	Cisplatin-based	4.29	(0.79, )		21.55	(0.78, )	

Multivariate analysis for PFS revealed that female sex (hazard ratio (HR) = 0.276; 95% CI = 0.131-0.581; p = 0.001), and normal lymphocyte counts at baseline (HR = 0.29; 95% CI = 0.14-0.626; p = 0.001) were an independent prognostic factors for longer PFS, while female sex (HR = 0.224; 95% CI = 0.079-0.635; p = 0.005) was the only prognostic factor for better overall survival.

## Discussion

This study characterizes the outcomes and prognostic factors of metastatic GC/GEJ adenocarcinoma treated at a tertiary center in Saudi Arabia. The median age at diagnosis in our patients was 52 years, which is younger than most reported literature from the Middle East, the far East, and the Western Hemisphere, where the median age in the latter is around 65 years [3,8-13]. This might be explained partially by the younger population of Saudi Arabia; however, other possible factors need to be sought. The male-to-female predominance was also less evident in our patient population than in reports from different parts of the world, where the ratio is approximately 1.8:1 [3,8-11].

The rate of GEJ as a primary disease site was 19.5%, comparable to the Eastern Hemisphere [8,14]; however, slightly less than the reported GEJ rate in the Western Hemisphere [9,15,16]. GEJ cancer is related to the higher incidence of gastroesophageal reflux disease (GERD) and obesity [17]. It is expected that GEJ cancer will increase in the Saudi population as the rate of obesity is increasing, as has been reported [18]. In our study, not all patients were tested for HER-2 status. Out of 28 patients tested, only four were HER-2 overexpressed by IHC, representing 9.8%. This represents one of the lowest reported HER-2 positivity in patients with metastatic GC [19]. Patients with treated HER-2-positive GC have improved survival compared to the HER-2-negative group. This might be one of the factors that resulted in a low PFS in our patient cohort.

The ORR in our patient’s cohort was lower than that reported in previous trials, with an ORR of 14.6% [20]. This can be explained due to more patients with PS of 2-4 in our study compared to clinical trials.

The median PFS in our study was disappointingly low at 4.1 months compared to reported data in the literature [10,14,20]. This inferior PFS can likely be explained by a higher percentage of patients with ECOG-PS of 2-4, accounting for 39% of our patient cohort [8,10,16]. The low rate of second-line treatment in our patient cohort of 36% also suggests a group of patients with poor characteristics, making them unfit for second-line therapy. In recent trials, second-line therapy has generally been delivered to 40-55% of patients receiving first-line treatment [21,22].

The median OS was 15.4 months, with a five-year survival of 39% vs. 12% in previous population-based survival in Saudi Arabia [23]. Whether the younger age of our patient cohort (median = 52 years) or the small sample size played a role in the higher survival rate is not entirely clear [3,9,10,24]. We were unable to obtain the dose intensity of chemotherapy in our patients. This is also an issue of debate where some trials have documented similar survival in elderly frail patients with poor PS with up to 40% dose reduction with oxaliplatin-based chemotherapy [25].

Multiple reports in the literature have studied the potential prognostic factors for metastatic gastric/GEJ cancer. In our study, the female sex was associated with significantly better PFS and OS by univariate and multivariate analysis, similar to other reports [3,9]. Additionally, normal lymphocyte count was associated with significant improvement in PFS in both univariate and multivariate analysis. This is comparable to reports confirming low neutrophil-to-lymphocyte ratio (NLR) associated with better PFS [26,27]. Patients with low NLR are expected to have a normal-high lymphocyte count.

The retrospective nature of our research, the small number of our cohort, and the lack of chemotherapy dose intensity are the main limitations of our study; however, it is the most extensive series from Saudi Arabia. Every effort should be made to improve on the current results. This would be achieved by early diagnosis and accessibility of patients to therapy and using a modern regimen containing immune checkpoint inhibitors.

## Conclusions

Metastatic GC in our part of the world continues to have poor outcomes. Despite the incorporation of immune checkpoint inhibitors lately as a new standard of care in the first-line setting, the outcome continues to be poor and warrants further research. Female sex and normal baseline lymphocyte count were independent prognostic factors associated with a better PFS, while female sex was an independent factor for better OS in metastatic GC patients.
